# Iron Tailings as Mineral Fillers and Their Effect on the Fatigue Performance of Asphalt Mastic

**DOI:** 10.3390/ma17122927

**Published:** 2024-06-14

**Authors:** Yaning Cui, Chundi Si, Song Li, Yanshun Jia, Bin Guo

**Affiliations:** 1 School of Traffic and Transportation, Shijiazhuang Tiedao University, Shijiazhuang 050043, China; cuiyaning@stdu.edu.cn (Y.C.); jiaysh@stdu.edu.cn (Y.J.); 2202206011@student.stdu.edu.cn (B.G.); 2 Key Laboratory of Traffic Safety and Control of Hebei Province, Shijiazhuang 050043, China; 3 State Key Laboratory of Mechanical Behavior and System Safety of Traffic Engineering Structures, Shijiazhuang 050043, China

**Keywords:** iron tailings, asphalt mastic, fatigue performance, interaction ability, interfacial adhesion energy

## Abstract

Incorporating iron tailings (ITs) into asphalt represents a new method for waste-to-resource conversion. The objective of this study is to evaluate the fatigue performance of ITs as fillers in asphalt mastic and investigate the interaction and interfacial adhesion energy between asphalt and ITs. To achieve that, the particle size distributions of two ITs and limestone filler (LF) were tested through a laser particle size analyzer; the morphology and structure characteristics were obtained by scanning electronic microscopy (SEM), the mineral compositions were conducted through X-ray diffraction (XRD), and the chemical compositions were tested through X-ray Fluorescence Spectrometer (XRF). Furthermore, the fatigue properties of asphalt mastic and the interaction between asphalt binder and mineral fillers (ITs and LFs) were evaluated by Dynamic Shear Rheometer (DSR). The interfacial adhesion energy between ITs and asphalt binder were calculated through molecular dynamics simulation. In the end, the correlation between the test results and the fatigue life is established based on the gray correlation analysis, the environmental and economic benefits of iron tailings asphalt pavement are further evaluated. The results show that the particle size distribution of ITs is concentrated between 30 μm and 150 μm, and the main component is quartz. ITs have rich angularity and a higher interaction ability with asphalt. The adhesion energy of iron tailings filler to asphalt is less than that of limestone. The correlation degree of the interfacial adhesion energy and interaction between asphalt and mineral filler with asphalt mastic fatigue life is close to 0.58. Under the combined action of interaction ability and interfacial adhesion energy, the fatigue life of IT asphalt mastic meets the requirements. ITs as a partial replacement for mineral fillers in asphalt pavement have great environmental and social effectiveness.

## 1. Introduction

ITs are the main solid by-product of iron ore mining and refining, which generally accounts for 60~80% of total ore [1]. From a global perspective, approximately 20–25 billion tons of mining waste are produced by the mining industry every year [2,3]. With the continuous development of the economy, the mining tailings have become the largest solid waste in China. The total amount of IT was reported to be approximately 39% among all tailings, including various metallic and nonmetallic tailings [4], the storage volume of ITs has exceeded 5 billion tons and is increasing at a rate of more than 300 million tons per year [5,6]. Therefore, the environmental and safety problems of tailings storage are becoming increasingly prominent. The construction and operation of tailings ponds not only require a large amount of labor, material resources, and financial resources but also pollute the environment, occupy a large amount of land resources, form huge safety hazards, and seriously threaten the safety of people’s lives and property. Iron tailings are generally fine in size. If they are used as a substitute for mineral filler, it will not only reduce construction costs but also greatly utilize iron tailings and create high economic and social benefits.

The construction of asphalt pavement requires a large amount of mineral fillers, and the mineral fillers are used to produce asphalt mastic. Asphalt mastic performance indirectly affects asphalt pavement service performance. Wei et al. [7,8] found that incorporating iron tailings into asphalt binder is beneficial for improving the high-temperature performance of asphalt mastic, which has enormous environmental benefits. Bastidas-Martinez et al. [9] indicate that iron tailing sands have little effect on the performance of a hot mix asphalt mixture and can meet the road performance requirements. Cao et al. [10,11] pointed out that the iron tailings asphalt mixture has poor crack resistance, but good rutting resistance and the incorporation of a silane coupling agent can improve the crack resistance of the iron tailings asphalt mixture. ITs asphalt mixture should also ensure their good durability and fatigue properties for promotion and application. As we all know, fatigue cracks of the asphalt mixture usually occur at the asphalt mastic or the asphalt–mineral interface [12]. However, there is little research on ITs asphalt mastic in this area.

In order to study the fatigue properties of asphalt mastic, some studies use storge (G′), loss modulus (G″), and loss tangent (tanδ) to evaluate fatigue resistance. However, the small loading strain of the sample cannot represent the actual destructive resistance of the material [13]. To solve this problem, the researchers invented the liner amplitude sweep (LAS) test, which not only greatly saves the test time of a fatigue test, but also has a strong correlation between the evaluation results and the field fatigue crack data [14,15].

Meanwhile, asphalt mastic fatigue behavior is affected by many factors, especially the interaction ability of asphalt–mineral fillers and their interfacial adhesion energy. Previous studies found that the interfacial phase capacity depends on the interaction capacity of asphalt and mineral fillers; the rheological properties and mechanical behavior of asphalt mastic affect the bonding and adhesion ability of asphalt mastic [16,17]. The index of the asphalt filler interaction ability is mainly constructed based on rheological parameters such as complex viscosity, complex modulus G*, and phase angle δ. The influence of energy dissipation, rheological properties of the composites, component concentration, interfacial tension, particle size of the fillers, and other factors were considered when studying the modulus enhancement effect of inorganic fillers on elastic composites [18]. A comprehensive evaluation index of the interaction capacity based on the modulus G* and phase angle δ was proposed to characterize the interaction capacity, which was defined as K.D.Ziegel-B-G* and K.D.Ziegel-B-δ. The two indicators are highly sensitive to the characteristics of mineral fillers [19].

In order to evaluate the adhesion energy between asphalt and ITs at the molecular scale, the interfacial adhesion energy was calculated by establishing an asphalt–aggregate molecular layer [20]. It was found that the aggregate surface had an important impact on the adhesion energy between asphalt and aggregates [21]. The adhesion ability depends on the mineral types and the orientation of the crystal face [22,23]. The interfacial adhesion energy between ITs and asphalt needs to be further studied.

**Figure 1:**
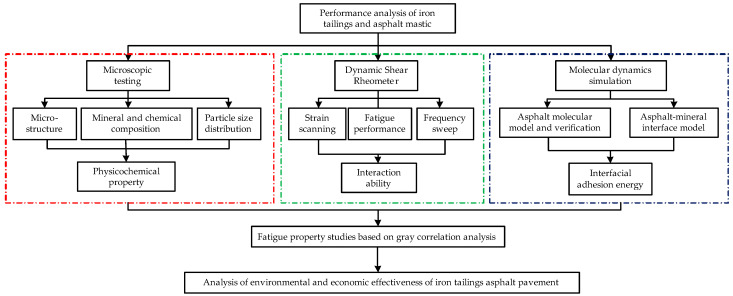
Research flowcharts.

On the basis of the above-mentioned consideration, this study investigates the particle size distribution, micromorphology, and mineral and chemical compositions of ITs first. The fatigue performance of asphalt mastic was investigated through the LAS test, and the interaction ability and interfacial adhesion energy between asphalt and ITs were further studied; meanwhile, their correlation degree with asphalt mastic life was established through a gray correlation analysis. Finally, the environmental and economic effectiveness of ITs applied to asphalt pavement are evaluated. The results would be beneficial for the efficient utilization of ITs and the long-term performance of asphalt pavement with ITs. The research flowchart is shown in Figure 1.

## 2. Materials and Methods

### 2.1. Materials Properties

The 70# neat asphalt binder applied in this study was tested according to JTG E20-2011 [24]. The results are shown in Table 1.

**Figure 2:**
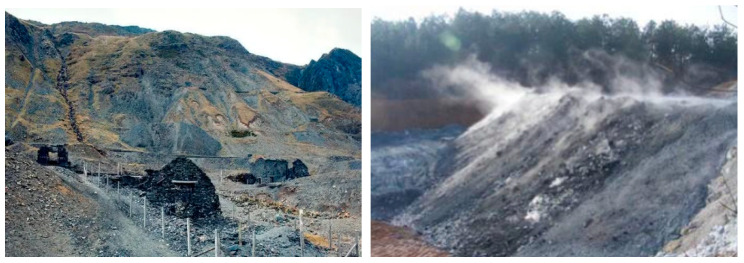
IT-stacking dam.

At the same time, three types of fillers were selected to prepare the asphalt mastics. IT fillers were simplified as IT-1 and IT-2. The IT in this study came from Shijiazhuang Hebei province, China. A large number of IT wastes were stacked in this area, which occupied a large amount of farmland and caused environmental pollution. Figure 2 shows the stacked ITs.

In this study, the ITs were passed through a 0.075 mm sieve to maintain the same initial distribution character as LFs. The basic properties of the three fillers were tested according to JET E42-2005 [25], as shown in Table 2. The hydrophilicity coefficient is used to evaluate the adhesion performance between the mineral filler and the asphalt. It is the ratio of the volume of mineral filler expanded in polar media to the volume expanded in nonpolar media. The larger the hydrophilicity coefficient, the better the adhesion performance between the mineral filler and the asphalt.

**Figure 3:**
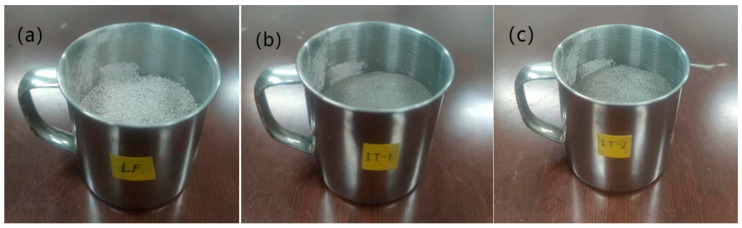
Three types of mineral fillers: (**a**) LF; (**b**) IT-1; and (**c**) IT-2.

It can be seen that the density of ITs is higher than LFs, which will affect the volume fraction of the filler applied in the asphalt mastic. The hydrophilic coefficient and water content of ITs are also higher than LFs, implying poorer adhesion between ITs and asphalt. To decrease the effect of free water on the performance of asphalt mastic, the fillers were dried to a constant weight before use. The LFs and ITs are shown in Figure 3.

### 2.2. Preparation of Asphalt Mastics

**Figure 4:**
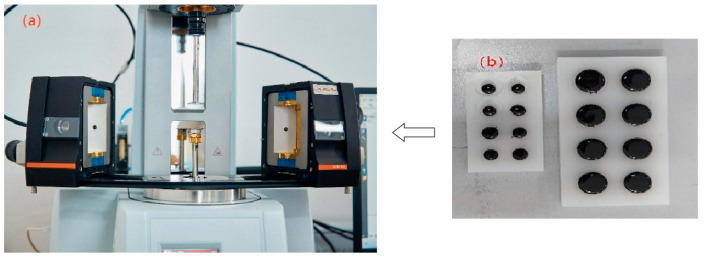
DSR equipment and experimental samples: (**a**) DSR; and (**b**) asphalt mastic samples.

This study selected a 70# neat asphalt binder and three kinds of mineral fillers to make the limestone asphalt mastic (LAM) and IT asphalt mastic (IAM) according to the design specification of the Superpave asphalt binder proposed by SHRP [26]. The f/a ratio of this study was 1.0. The details of the preparation process are shown below. Firstly, the mineral filler was passed through the standard sieve of 0.075 mm, then it was placed in the 150 °C oven and dried for 2 h until the mass was constant; at the same time, the 70# base asphalt was put in the oven for heating for 2 h at 135 °C. Second, the mineral fillers were incorporated into the asphalt binder gradually many times, while the high-speed shear instrument was used to mix the asphalt mastic at 135 °C. The mastics were cut for 30 min at a shear rate of 1000 r/min uniformly. To prevent mineral filler precipitation, it must be re-stirred before each experiment. The DSR equipment and experimental samples can be seen in Figure 4.

## 3. Experimental Methods

### 3.1. Physicochemical Properties of ITs and LFs

The particle size distribution of ITs and LFs was analyzed by a laser particle size analyzer. By converting some quantified scattering data through a suitable optical model and mathematical process, the particle size volume relative to the total volume is obtained. The particle size distribution is the key factor that affects the interaction ability of asphalt and minerals. The mineralogical compositions of these aggregates were then investigated by XRD analysis with a scanning angle from 3° to 90°. In this way, the microscopic mineral molecular model can be obtained. Finally, SEM was used to characterize the microstructure of the mineral fillers. The interaction ability of asphalt and minerals can be predicted by micromorphological analysis.

### 3.2. Fatigue Properties of Asphalt Mastic

The fatigue test (LAS) of asphalt mastic with different mineral fillers was carried out with DSR. The LAS test is a kind of experiment to characterize the fatigue properties of asphalt based on the theory of viscoelastic continuum damage (VECD). A LAS test must perform a frequency scanning experiment to obtain damage analysis parameters first. The frequency is scanned from 0.2 Hz to 30 Hz, and the strain level is set at 0.1%. In the amplitude scanning, the temperature was set at 25 °C, the loading amplitude increased linearly from 0.1% to 30% at the loading frequency of 10 Hz, and the loading time was 310 s. In the VECD model, the integrity parameter *C* and the cumulative asphalt damage parameter are introduced to characterize the process of asphalt fatigue damage. When *C* = 1, asphalt is in an undamaged state, and when *C* = 0, asphalt is damaged. The complex modulus G*, phase angle δ, storage modulus G*cosσ, loss modulus G*sinσ, share stress σ, and strain ℇ are recorded for each cycle. The cumulative damage parameter *D* was used to characterize the fatigue damage process of asphalt samples, as shown in the following equation:

(1) $$D(t)≅\sum_{i=1}^{N}\left[\pi {\gamma_{0}}^{2}(C_{i-1}-C_{i}\right){]}^{\frac{\alpha }{1+\alpha }}{\left(t_{i}-t_{i-1}\right)}^{\frac{1}{1+\alpha }}$$

where *C_t_* = G*(t)/G*, *C_t_* is the integrity parameter that is the applied strain, %, G* is the complex shear modulus, *t* is time, and *α* is the coefficient, which is obtained using the following formula:

(2) $$\log(\left|G^{*}\right|\cos\delta )=m(\log\omega )+b$$

(3) α=1+1/m

where *m* is the slope of the logarithmic curve between the storage modulus and the applied frequency, which is obtained by the fitting curve. The relationship between the integrity parameter *C* and the cumulative damage parameter *D* is established, as shown in Equation (4),

(4) $$C(t)=C_{0}-C_{1}{\left(D\right)}^{C_{2}}$$

where *C*_0_ = 1, and *C*_1_ and *C*_2_ are the fitting parameters, which can be calculated using the following formula:

(5) $$\log(C_{0}-C(t))=\log(C_{1})+C_{2}\cdot \log(D)$$

It is assumed that when fatigue damage occurs in the sample, *D*(*t*) is expressed by *D_f_*, and the criterion of fatigue failure is that the integrity parameter *C*(*t*) decreases to 35%. *D_f_* can be obtained from the following formula:

(6) $$D_{f}=(0.35){\left(\frac{C_{0}}{C_{1}}\right)}^{\frac{1}{C2}}$$

(7) $$A_{35}=\frac{f{\left(D_{f}\right)}^{k}}{k{\left(\pi C_{1}C_{2}\right)}^{\alpha }}$$

(8) $$N_{f}=A_{35}{\left(\gamma_{\max}\right)}^{B}$$

In this formula, *f* is the loading frequency, 10 Hz, and *B* = 2*α*. *γ*_max_ is the maximum expected strain of asphalt under the pavement structure.

### 3.3. Interaction Ability of Asphalt Binder and Mineral Fillers

The strain sweep analysis of asphalt and asphalt mastic was conducted from 0.01% to 100% at 10 Hz and 25 °C. Then, the frequency scanning test was conducted from 0.01 Hz to 10 Hz dynamic frequency at 0.1% strain and 25 °C. The linear strain range, complex modulus, and phase angle of asphalt and asphalt mastic were obtained by the above two experiments. The interaction behavior of the asphalt binder and mineral filler directly affects its viscoelastic properties and mechanical behaviors. The particle size, distribution state, acidity, and alkalinity of minerals are important factors that affect the interaction ability with asphalt [27]. As mentioned above, the K-B-G* and *K-B-δ* indexes are suitable to characterize the interaction capacity of asphalt and mineral filler. The definition and calculation formulas are as follows:

(9) $$K-B-G^{*}=\frac{(G_{c}^{*}/G_{m}^{*})-1}{(1.5+G_{c}^{*}/G_{m}^{*})\times \phi_{f}}$$

(10) $$K-B-\delta =\frac{(\tan\delta_{m}/\tan\delta_{c})-1}{1.5\times \phi_{f}}$$

(11) $$\phi_{f}=\frac{V_{f}}{V_{a}+V_{f}}$$

where K-B-G* and *K-B-δ* are the interaction capability evaluation indexes based on the complex modulus and phase angle; *G*_c_^*^ is the complex modulus of the asphalt mastic; *G*_m_* is the complex modulus of the asphalt binder; *δ*_c_ is the phase angle of the asphalt mastic, *δ*_m_ is the phase angle of the asphalt binder; *φ_f_* is the volume fraction of the mineral filler; *V_f_* is the volume of the mineral filler; and *V_a_* is the volume of the asphalt.

### 3.4. Interfacial Adhesion Energy of Asphalt Binder and Mineral Fillers

In order to establish the correlation degree between interfacial adhesion energy and fatigue performance of asphalt mastic. The asphalt molecular model was established according to the past investigation [28]; the model was verified by the parameters of asphalt density, radial distribution function, cohesion energy density, and solubility parameter, and the results were obtained. Then, the mineral components of limestone and ITs were established based on the XRD test. The final asphalt–mineral interface model was established to analyze the interfacial adhesion energy between asphalt and different minerals by calculating the molecular dynamics.

The interfacial adhesion energy is a physical quantity that measures the interaction between molecules of two different phases. It is the energy that reaches a stable state after mixing various immiscible substances. The negative energy means that substances attract each other. In this case, the greater the absolute value, the stronger the attraction between substances. The interfacial adhesion energy is calculated according to the following formula:

(12) $$E_{interface}=E_{total}-(E_{asphalt}+E_{mineral})$$

(13) $$\gamma =\frac{E_{interface}}{A}$$

where *E_interface_* is the interfacial adhesion energy; *E_total_* is the total cohesive energy of the mixture system of the asphalt and mineral; *E_asphalt_* is the cohesive energy of the asphalt; *E_mineral_* is the cohesive energy of the mineral; and *A* is the interface area.

### 3.5. Gray Correlation Analysis of Influencing Factors of Fatigue Performance

The influence of various factors on fatigue life can be analyzed using the gray correlation analysis method. Gray correlation analysis is a statistical analysis method to evaluate the degree of influence of multiple factors on the system, and it is used to determine the primary and secondary factors that affect the system. This method quantifies the correlation degree between many factors. A high correlation degree indicates that the relative change law between the factors is consistent; on the contrary, there is no obvious influence between the factors. The calculation steps of the correlation degree are as follows:

(1) Establish and initialize the comparison series and reference series.
  (14) $$X_{0}=\left\{x_{0}(K),\;K=1,\;2,\;3,\;\cdots ,\;n\right\}$$
  (15) $$X_{i}=\left\{x_{i}(K),\;K=1,\;2,\;3,\;\cdots ,\;n\right\},\;i=1,\;2,\;3,\;\cdots ,\;m$$
  In the formula, *X*_0_ and *X*_i_ are reference series and comparison series, respectively.
(2) Divide the first value of each sequence by the other values to obtain a new sequence:
  (16) $$Y_{0}=\left\{y_{0}(K),\;K=1,\;2,\;3,\;\cdots ,\;n\right\}=\left\{\frac{x_{0}(K)}{x_{0}(1)},\;K=1,\;2,\;3,\;\cdots ,\;n\right\}$$
  (17) $$Y_{i}=\left\{y_{i}(K),\;K=1,\;2,\;3,\cdots ,\;n\right\}=\left\{\frac{x_{i}(K)}{x_{i}(1)},K=1,\;2,\;3,\;\cdots ,\;n;\;i=1,\;2,\;3,\;\cdots ,\;m\right\}$$
  where *Y*_0_ and *Y*_i_ are the reference series and the comparison series after initializing.
(3) Calculate the difference series and correlation coefficients between the difference series and the correlation coefficient and correlation degree:
  (18) $$\Delta_{i}(K)=\left|y_{0}(K)-y_{i}(K)\right|$$
  (19) $$\xi_{i}(K)=\frac{{\left[\Delta_{i}(K)\right]}_{\min}+\rho {\left[\Delta_{i}(K)\right]}_{\max}}{\Delta_{i}(K)+\rho {\left[\Delta_{i}(K)\right]}_{\max}}$$
  (20) $$\gamma_{i}=\frac{1}{n}\sum_{K=1}^{n}\xi_{i}(K)$$
  where Δ*_i_*(*K*) and ξ*_i_*(*K*) are the difference sequence and the correlation coefficient between the reference series and the comparison series after initializing; *ρ* is the resolution coefficient, and the value is between 0 and 1, usually *ρ* = 0.5; [Δ*_i_*(*K*)]_max_ and [Δ*_i_*(*K*)]_min_ are the maximum and minimum values of the difference sequence; and *γ*_i_ is the correlation degree.

## 4. Results and Discussions

### 4.1. Physicochemical Properties of ITs and LFs

#### 4.1.1. Particle Size Distribution

**Figure 5:**
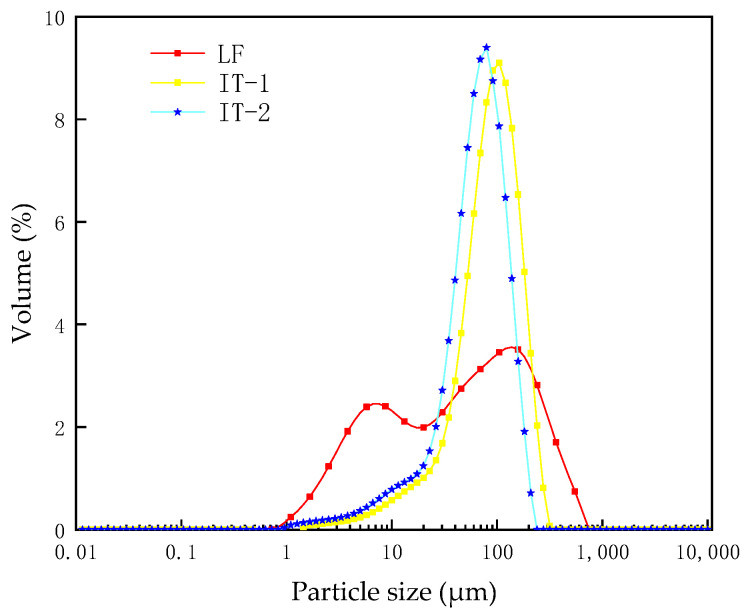
Distribution of particle size.

The particle size distribution can be seen in Figure 5.

Figure 5 shows the particle size distribution of limestone and two types of iron tailing fillers. It can be seen from the figure that the particle size of iron tailing filler is mainly distributed between 30 μm and 150 μm, accounting for 83%, and the distribution is uneven. The particle size of IT-2 is slightly smaller than that of IT-1; the particle size span of limestone mineral filler is relatively large, mainly distributed between 5 μm and 300 μm, accounting for 82%, and the distribution is relatively uniform. In terms of the 5 μm~30 μm part, limestone filler accounts for 30%, and IT accounts for about 12%; the proportion of ultra-fine powder in limestone is about three times that of iron tailings. On the whole, the limestone mineral filler is finer than the IT filler and has a higher contact area with asphalt [29].

#### 4.1.2. Micromorphology

**Figure 6:**
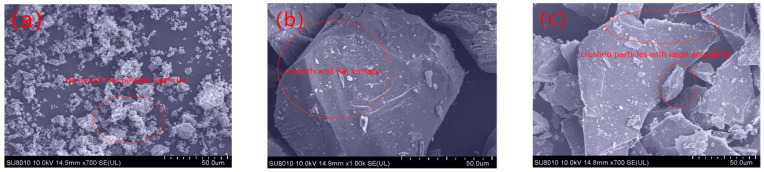
SEM of three fillers: (**a**) LF; (**b**) IT-1; and (**c**) IT-2.

The exterior morphology and crystalline structure of LFs and ITs were compared using scanning electron microscopy (SEM) analysis. The results are shown in Figure 6.

Figure 6 shows that limestone mineral filler is finer and smoother, resulting in higher asphalt absorption on its surface, since a smaller particle size gives more surface area to adsorb more bitumen molecules. Meanwhile, the IT particles have larger particle sizes and angular shapes which may result in higher friction and a stronger interaction with asphalt. When comparing the two kinds of IT, the surface of IT-1 is smooth and flat; however, IT-2 is broken and the angular shape of IT-2 is more abundant, and it can be inferred that the interaction between IT-2 and asphalt is higher [30].

#### 4.1.3. Mineral Composition

**Figure 7:**
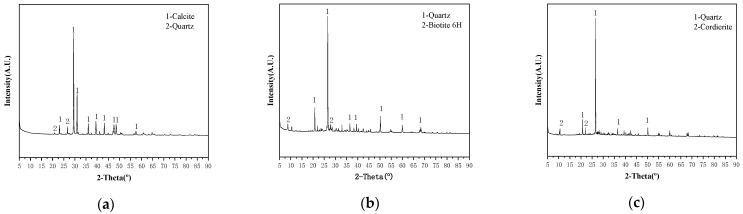
Mineral composition of three fillers: (**a**) LF; (**b**) IT-1; and (**c**) IT-2.

The XRD diffractograms of the three fillers in this study can be seen in Figure 7.

According to Figure 7, there is a significant difference in the mineral composition between limestone and iron tailings sand. The predominant composition of LFs is calcite, which makes the limestone filler alkaline. Silica was found primarily in the form of quartz in IT-1 and IT-2. The high amount of silica in fillers is commonly associated with poor moisture sensitivity and poor adhesion to asphalt, and it is undesirable for the asphalt mixture [30].

#### 4.1.4. Chemical Composition

**Figure 8:**
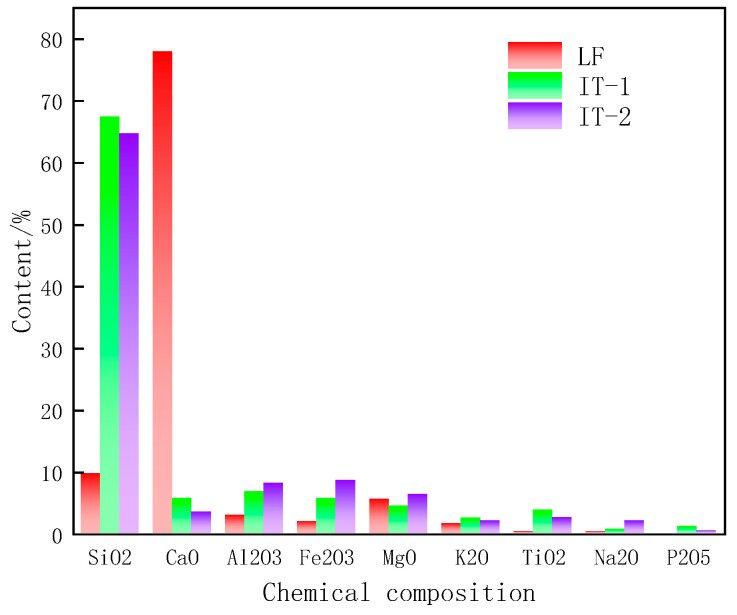
Chemical composition of three fillers.

Figure 8 shows the chemical composition of the three mineral fillers. The main chemical component of limestone filler is CaO, and the main chemical component of the two iron tailings filler is SiO_2_, occupying about 68%, so the ITs are acidic [29]; the content of Al_2_O_3_ and Fe_2_O_3_ in iron tailings filler is slightly higher than that of limestone filler. Mineral composition analysis and chemical composition analysis lay the foundation for the subsequent establishment of the mineral molecular model.

### 4.2. Fatigue Performance Analysis of Asphalt Mastic

Through the LAS test, the stress–strain curve of the asphalt mastic is shown in Figure 8.

**Figure 9:**
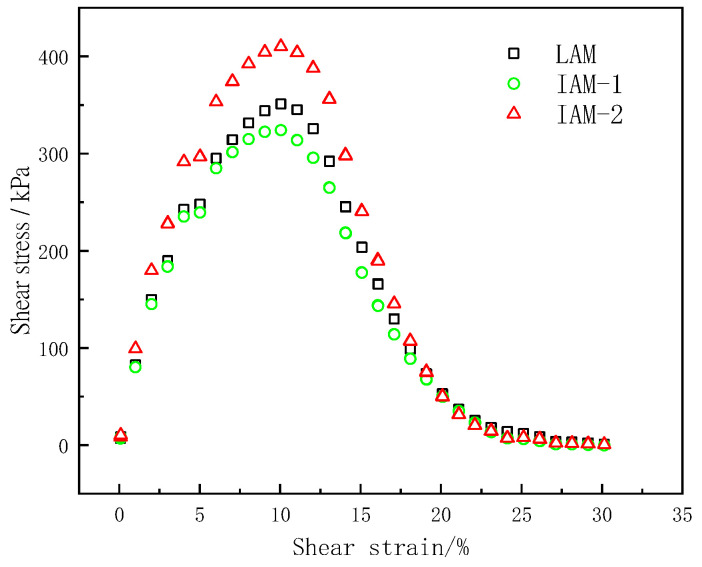
Stress–strain curve of asphalt mastic.

In the LAS test, the shear stress of the asphalt mastic sample has a peak value, and the shear stress inflection point is caused by the failure of the asphalt mastic sample due to the increase in the shear strain. The strain at the peak stress in the stress–strain curve can be used as an evaluation index to evaluate the fatigue life of the asphalt mastic. When the strain reaches 8%, the three samples show obvious damage, but the corresponding stress peaks are different. It can be seen from Figure 9 that IAM-2 can withstand greater stress than the other two asphalt mastics under the same strain, and IAM-2 has the highest strain sensitivity. In the peak region, the shear stress of asphalt mastic is not sensitive to changes in strain but can maintain a high level. After exceeding the maximum stress, the stress value decreases with increasing strain, indicating that the asphalt mastic is damaged. The stress–strain curve can only qualitatively predict the influence of mastic on fatigue life, and it is necessary to investigate the fatigue properties and other indicators thoroughly in the LAS test.

**Figure 10:**
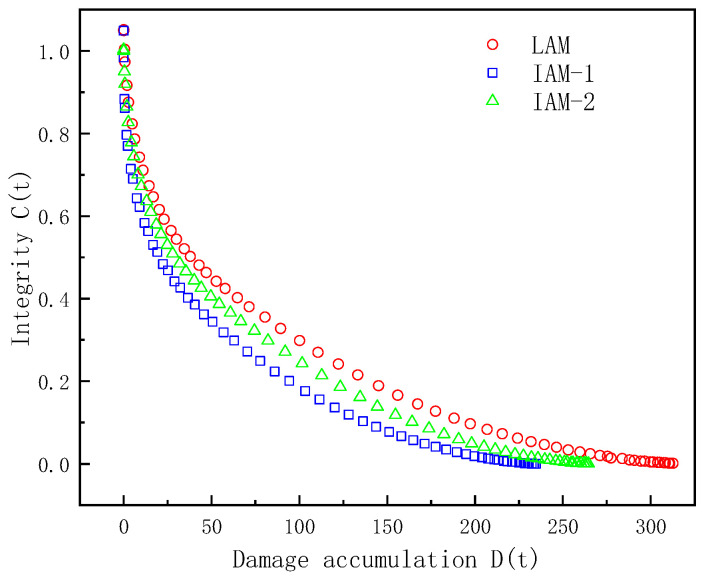
Analysis of cumulative damage to asphalt mastic.

The fatigue damage curves of the asphalt samples were plotted using the VECD model. The relationship between the integrity index *C*(t) and the cumulative damage *D*(t) in the LAS test is shown in Figure 10.

In Figure 10, the horizontal axis *D*(*t*) represents the cumulative damage parameter, and the vertical axis *C* represents the integrity parameters of the asphalt mastic samples. From the slope rate of the fatigue damage curve, it can be seen that the virtual modulus of the IMA-1 asphalt mastic sample decreases rapidly, with the damage speed being the fastest. Under the same cumulative damage, the integrity of IAM is slightly lower than that of LAM, indicating that LAM has a better anti-fatigue performance than IAM. The integrity of IAM-1 decreases faster with increasing cumulative damage, and under the same cumulative damage, the integrity of IAM-2 is better than IAM-1. This is because IT-2 has various angularities and better adhesion to asphalt, so IAM-2 has a better anti-fatigue performance than IAM-1. The parameters of the calculated VECD model are shown in Table 3.

In Table 3, α represents the relationship between the storage modulus and the frequency, and because the strain during frequency scanning is small, each asphalt mastic sample is in an undamaged state; therefore, the difference between the asphalt mastic is not much different. *C*_1_ and *C*_2_ reflect the relationship between the virtual modulus G* sinδ and the cumulative fatigue failure variable *D*(t). It can be found that the coefficient of variation of the test results for both parameters α and B is small, and parameter A is calculated using damage model parameters *C*_1_ and *C*_2_, and the IT variability is large. The higher the A value, the longer the fatigue life of asphalt mastic under 5% strain. The higher the absolute value of B, the higher the sensitivity of the asphalt mastic to the strain level [31].

**Figure 11:**
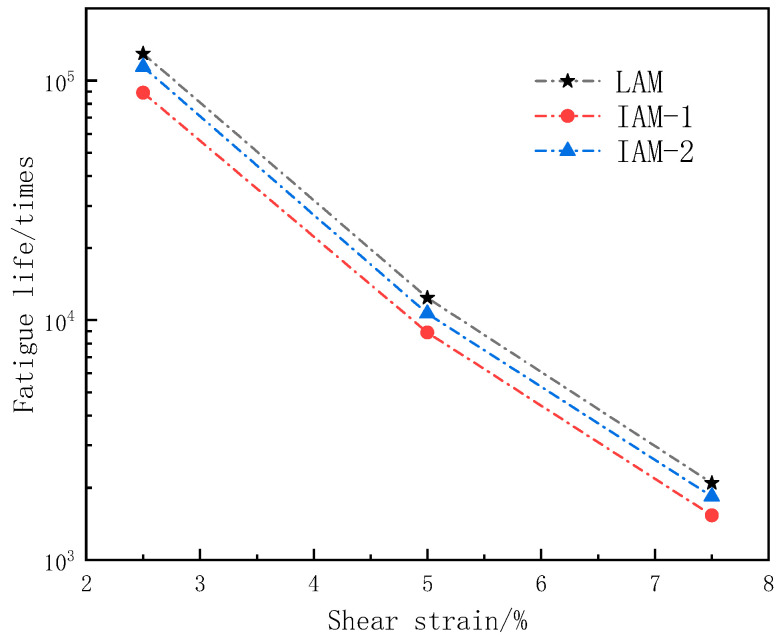
Asphalt mastic fatigue life under different shear strain conditions.

Figure 11 shows the fatigue life of three types of asphalt mastics under three strain conditions (2.5%, 5.0%, 7.5%).

As can be seen from Figure 11, the fatigue life of asphalt mastic prepared from limestone filler is better than that of ITs filler. LAM presents the largest fatigue life under the three strain conditions, and IAM-1 has the lowest fatigue life. With the increase in strain level, the fatigue life of asphalt mastic decreases sharply, but the difference in fatigue life of the three kinds of asphalt mastic is not great.

**Figure 12:**
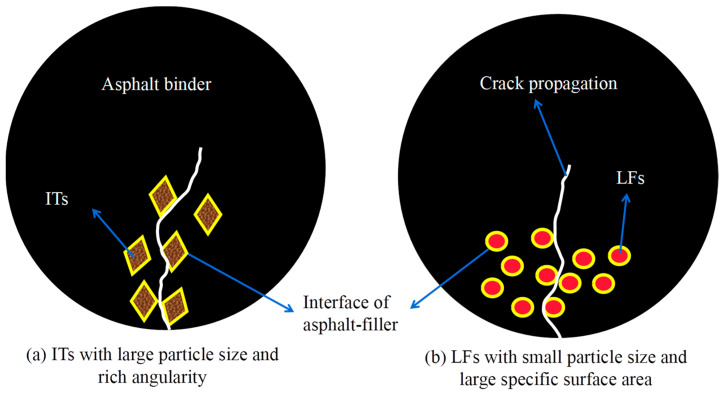
Forms of fatigue failure among asphalt mastic in LAS test.

Figure 12 shows the fatigue failure mode of asphalt mastic prepared using fillers with different particle sizes and specific surface areas under the same filler-to-binder ratio.

As shown in Figure 12, in the LAS fatigue test progress, the asphalt and filler interface is destroyed, and with an increasing number of particle fillers, the crack propagation path becomes longer and wider. Therefore, the filling of the particles is beneficial to improve the fatigue life of asphalt mastic. However, particle filling will cause asphalt hardening, increase the sensitivity of fatigue life to strain, and lead to a decrease in the fatigue life of asphalt mastic under large strain conditions. Therefore, the interaction between the effect of particle filling enhancement on fatigue life and ITs’ negative effect on strain sensitivity jointly determines the fatigue performance of asphalt mastic [32]. The specific surface area of LF is larger, and the contact interface with asphalt is wider; in addition, the surface shape of the IT particles is irregular and the edges and corners are rich, which is conducive to the adhesion of particles and asphalt. Both effects can improve the fatigue life of asphalt mastic. Therefore, it is necessary to analyze the interaction between the two fillers and asphalt.

It can be inferred that the fatigue life is affected by many factors, the physical and chemical properties of filler particles have a significant impact on the fatigue life of asphalt mastic, and it is necessary to analyze the correlation of each factor on the fatigue life. Therefore, the influence of the asphalt–mineral interaction ability and IT interface energy on fatigue properties was further explored, and the correlation between the influencing factors and fatigue life will be studied next.

### 4.3. Interaction Ability of Asphalt Binder and Mineral Fillers

In this section, the strain scanning of asphalt and mastic is performed. Figure 12 shows the strain scanning curves of three asphalt mastics.

**Figure 13:**
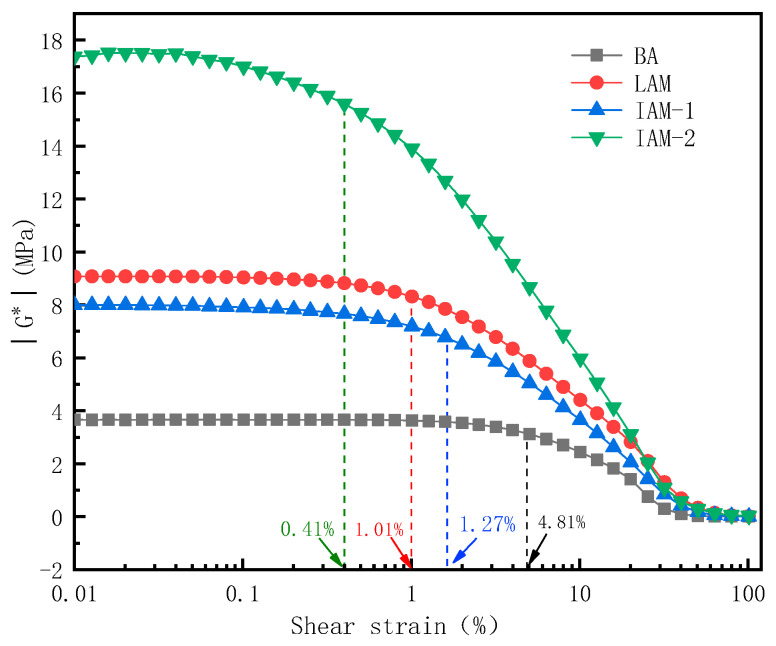
Strain scanning of each asphalt mastic.

The strain scanning test of each asphalt mastic is shown in Figure 12. As shown in Figure 13, the maximum strain values were obtained in the linear and nonlinear viscoelastic regions, and the linear viscoelastic zone of the asphalt mastic is smaller than asphalt. The critical strains in the linear viscoelastic range of asphalt binder, LAM, IAM-1, and IAM-2 are 4.81%, 1.01%, 1.27%, and 0.41%. Therefore, to analyze the interaction between asphalt and mineral filler in the linear range, the 0.1% strain value was used as the strain level in the next frequency scanning test. Under the same strain, the complex modulus of IAM-2 is the highest, indicating that the asphalt mastic is greatly affected by the microstructure of the filling particles; the complex modulus of LAM is higher than IAM-1. This is because, at the same mass ratio, the density of LF is smaller, resulting in a larger volume fraction and surface area. The larger specific surface area is conducive to adsorbing more asphalt, which will benefit the interaction with asphalt.

**Figure 14:**
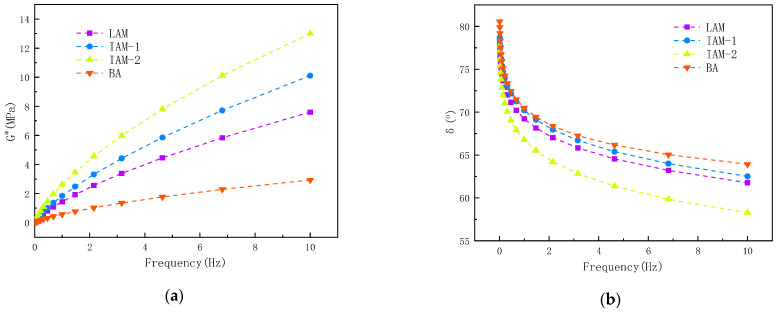
Frequency scanning of each asphalt mastic: (**a**) G*; and (**b**) δ.

Figure 14 shows the variation curves of the complex shear modulus (|G*|) and the phase angle (δ) of asphalt and asphalt mastic along with the loading frequency.

It can be seen in Figure 14 that the complex modulus of LAM, IAM-1, and IAM-2 are significantly higher than that of basic asphalt in the full frequency loading range, the modulus of IAM-2 is highest, and the two types of IAM complex modulus are both higher than LAM. On the contrary, the change in phase angle of the asphalt mastic is opposite to the change in the complex modulus. The phase angle of all asphalt mastic is smaller than that of base asphalt. The order of phase angle is BA > IAM-1 > LAM > IAM-2. An analysis shows that the incorporation of mineral filler enhances the elastic properties of asphalt mastic, weakens the viscoelastic hysteresis response of asphalt mastic, and reduces phase angle [33], and the modification effect of IAM-2 is the most significant.

**Figure 15:**
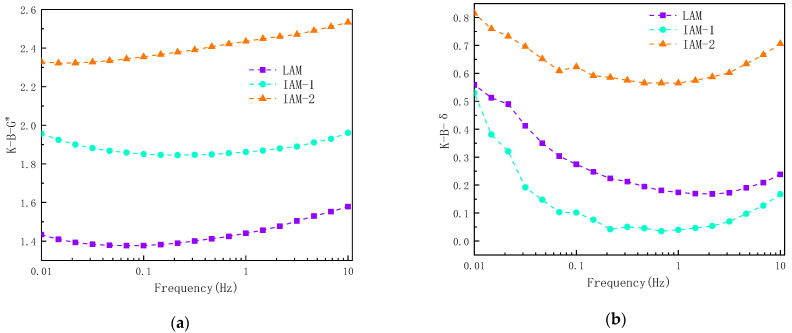
Asphalt–mineral filler interaction capacity at different loading frequencies: (**a**) K-B-G*; and (**b**) K-B-δ.

Based on the results of complex modulus and phase angle, this study investigated the interaction ability of ITs and asphalt, as shown in Figure 15.

The results found that K-B-G* shows an increasing trend with increasing loading frequency, indicating that the interaction ability of asphalt and mineral filler is affected by the loading frequency. It is obvious that the K-B-G* and K-B-δ values of IAM-2 are higher than those of LAM due to the complex angular properties of ITs. This may be due to the complex microstructure and particle size distribution of IT-2. In Figure 14b, it can be obviously seen that the K-B-δ value varies greatly with frequency, the interaction ability decreases first and then increases with frequency changes, and the index is smallest when the frequency is between 0.5 Hz and 5 Hz. As can be seen, it is not obvious to use K-B-δ to represent the interaction, so it is suggested to use K-B-G* to represent the interaction ability of asphalt and minerals.

### 4.4. Interface Energy of Asphalt Binder and Mineral Fillers

#### 4.4.1. Establishment of Asphalt Molecular Model

**Figure 16:**
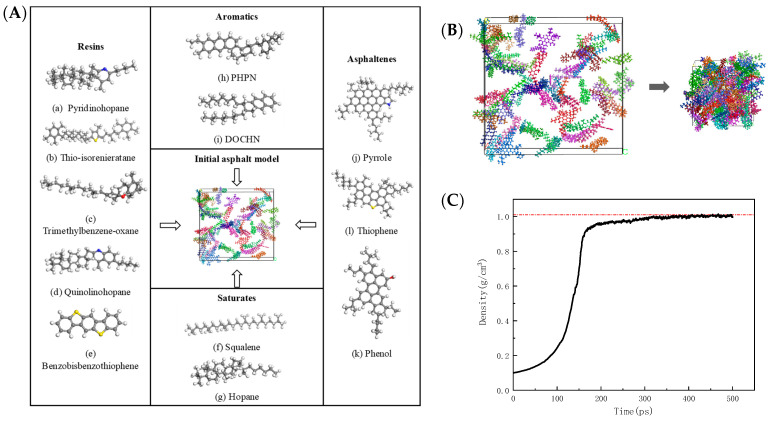
Molecular model of asphalt: (**A**) model compositions; (**B**) dynamic equilibrium; and (**C**) density variation in dynamic equilibrium.

The four-component separation method and 12 compounds were selected to represent the main components of 70# neat asphalt binder and the number of molecules of various compounds was determined according to the measured values of the elemental composition and the proportion component of asphalt in this study, as can be seen in Figure 16. In Figure 16A, 12 colors represent 12 asphalt components.

The density of the asphalt is obtained by calculating 500 ps under the NPT ensemble and the pressure condition of 1 atm; the density is an important physical and mechanical property of the asphalt and is often used to verify the reliability of the molecular models in MD simulations, with the optimization time reaching 500 ps, and the density values of the asphalt binder reached to 1.003 g/cm^3^as shown by the red line in Figure 16C.

#### 4.4.2. RDF Analysis of Asphalt Model

**Figure 17:**
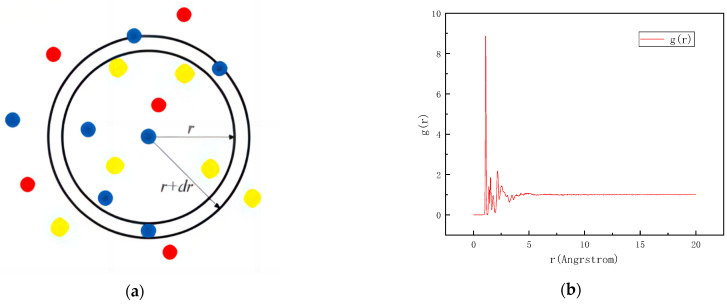
Asphalt RDF: (**a**) schematic diagram; and (**b**) RDF curve.

The radial distribution function (RDF) refers to the probability of finding a particle in the space at a certain distance from a given particle in the system, The radial distribution function can be used to study the order of matter to characterize the packing state of atoms and the distance between them. The RDF formula is as follows:

(21) $$g(r)=\frac{dN}{4\pi \rho r^{2}dr}$$

where *ρ* is the density (g/cm^3^); *r* is the distance between the particles (m); and *N* is the total number of particles. As can be seen from Figure 17, in the schematic diagram, different colors represent different types of particles, the curve is vibrating acutely within 4 Å; after 5 Å, the deformation is gentle and tends to 1, indicating the particles are disordered, the established model is a typical amorphous structure.

#### 4.4.3. Interfacial Adhesion Energy between Asphalt Binder and Mineral Fillers

**Figure 18:**
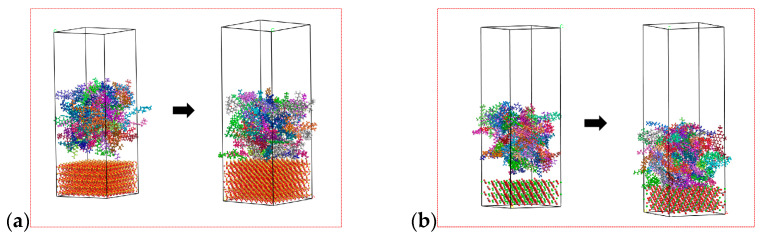
Interface adsorption model: (**a**) IT-asphalt; and (**b**) LF-asphalt.

In order to facilitate the model, the main components accounting for minerals are used to replace the total components. According to the test results of mineral composition and chemical composition, calcite (CaCO_3_) was selected as a representative mineral aggregate of limestone, and quartz (SiO_2_) was selected as the representative mineral aggregate of ITs, which has been adopted in the reference [11], however, ignoring the influence of other components will inevitably reduce the accuracy of the model. After geometric optimization and dynamic equilibrium, the asphalt–mineral interface layer model was established. The interface adsorption model before and after molecular dynamics calculation can be seen in Figure 18.

As shown in Figure 18, after dynamic equilibrium, the asphalt is adsorbed on the surface of the mineral fillers. The calculated results are shown in Table 4; the results show that the interfacial adhesion energy between asphalt and ITs is −0.176 kcal·mol^−1^·Å^−2^, much smaller than LF. However, due to the simplification of the molecular model of mineral components in the study, there are some errors in the calculated results.

### 4.5. Gray Correlation Analysis of Influencing Factors of Fatigue Performance

The gray correlation analysis method is used to establish the correlation degree between interfacial adhesion energy, interaction ability, complex modulus, and asphalt mastic fatigue life. The fatigue life is selected as the reference sequence, and the interfacial adhesion energy, interaction ability, and complex modulus are selected as the comparison sequence. Then, the gray correlation analysis is carried out, as shown in Table 5. The results of a dimensionless processing of variables are shown in Table 6. And the computed difference sequence is shown in Table 7.

The correlation between the degree of interfacial adhesion energy, interaction ability, and complex modulus with fatigue life is shown in Table 8. It can be seen in Table 8 that the correlation degree of interfacial adhesion energy, interaction index, and complex modulus is not much different with fatigue life. The three factors’ correlation degree with asphalt mastic fatigue life is nearly 0.58. This phenomenon shows that the fatigue life is affected by both physical and chemical factors, and the two factors have almost the same impact on the fatigue life, and so the final fatigue life of the three types of asphalt mastic is not much different. The micromorphology of IT-2 has rich edges and corners, resulting in a greater interaction with asphalt. These physical factors are conducive to increasing the fatigue life of asphalt mastic.

### 4.6. Effectiveness Analysis

In terms of society, pollutants caused by the improper disposal of ITs will spread through various routes and ultimately endanger human production, life, and health [34]. The global demand for fillers in the foreseeable future is going to be increased and as the movement towards more environmentally feasible pavement materials endures, it is necessary for the pavement industry to consider alternative materials that previously would not have been considered for the purpose. With the increasing awareness of environmental protection and resource depletion, the prospects of the market for the IT market are becoming more and more broad. The state’s support policy for the utilization of waste resources will provide more opportunities for the development and utilization of iron ore tailings resources. According to the statistics of the National Highway Department, the existing asphalt pavement in the country is 240 billion m^2^ (excluding rural roads).

Taking asphalt pavement maintenance as an example, iron tailings are applied to the maintenance of the pavement. If the amount of 200 g/m^2^ of iron tailings is used, 48 million tons of iron tailings can be eliminated in the country. The price of limestone is 40–45 yuan/ton. However, iron tailings are only 10–13 yuan/ton. For comparison, the price of iron tailings is approximately one-quarter of limestone. Assume that 14,992 t of iron tailings are deposited per hectare, 32,000 hectares of land can be saved and the service life of the asphalt pavement can be extended after maintenance for 2 years. If the cost is calculated as 52 yuan/m, each square meter of pavement can save 104 yuan, which can increase economic benefits by 244.8 billion yuan. Therefore, the recycling of a huge amount of ITs in highway infrastructure construction is undoubtedly a meaningful practice for the mining industry and the transportation industry.

## 5. Conclusions

The optimum utilization of ITs as alternative fillers has the potential to be beneficial for the asphalt pavement infrastructure as well as for the environment. This study uses multidisciplinary theories and multi-scale methods to investigate the physical and chemical properties of ITs; the interaction ability and interfacial adhesion energy between ITs and asphalt were studied, and their correlation degree on the fatigue performance of asphalt mastic was established. Based on test results and data analyses, the main conclusions can be summarized as follows:

1. The particle size distribution of IT is concentrated in 30 μm~150 μm with obvious edges and corners, and the microstructure of IT-2 has more angularity than the other two fillers, which will enhance the interaction ability between the asphalt binder and the mineral fillers. While the LF is uniformly distributed in 5 μm~300 μm, presenting cluster distribution, smaller particle sizes can adsorb more asphalt, which is also beneficial to improve the fatigue life of asphalt mastic.
2. The fatigue life of the two kinds of iron tailings asphalt mastic is not much different from that of limestone asphalt mastic. Among them, the fatigue life of LAM is the highest, and the fatigue life of IAM-2 is slightly better than that of IAM-1. ITs can be used in asphalt pavement instead of limestone fillers.
3. The main mineral composition of ITs is SiO_2_. ITs are acidic minerals, whose interfacial adhesion energy with asphalt is low. The main mineral composition of limestone is CaCO_3_, which is an alkaline mineral with higher interfacial adhesion energy. On the contrary, the interaction between ITs and asphalt is higher than that of LF based on the K-B-G*, which may be caused by the rich angularity of ITs.
4. Through gray correlation analysis, it was found that the adhesion energy and interaction ability between asphalt and mineral fillers jointly determined the asphalt mastic fatigue life. The correlation degree of interfacial adhesion energy and interaction ability with asphalt mastic fatigue life is almost the same, close to 0.58.
5. The application of ITs as a substitute for LF in asphalt pavement construction provides a new perspective on solid waste utilization. The fatigue performance of IAM meets the requirements, and it will create huge economic, environmental, and social benefits.

This paper only analyzes the fatigue performance of asphalt mastic and its correlation degree with other test results. It is suggested that the influence mechanism of interfacial adhesion failure between iron tailings and asphalt on the fatigue performance of asphalt mastic should be further studied, and the coupling effect of water and thermal field on the fatigue performance of iron tailings asphalt mastic is recommended.

## Figures and Tables

**Table 1:** Basic properties of 70# neat asphalt binder.

Test Parameter	Result	Technical Requirements
Density (g/cm^3^)	1.032	-
Penetration (25 °C, 0.1 mm)	62	60–80
Softening point (°C)	48	>46
Ductility (10 °C, cm)	75	>20
Viscosity (60 °C, Pa·s)	195	>180

**Table 2:** Basic properties of mineral fillers.

Properties	IT-1	IT-2	LF	Technical Requirements
Density (g/cm^3^)	2.95	3.02	2.77	>2.5
Hydrophilic coefficient (%)	0.79	0.82	0.22	<1
Water content (%)	1.2	1.5	0.21	<1

**Table 3:** LAS test parameters for asphalt mastic (20 °C).

Asphalt Mastic Type		Damage Model Parameter				Complex Fatigue Parameter		N_f_@5%
		C_0_	C_1_	C_2_	α	A	B	
LAM	average	1	0.1575	0.3335	2.192	1.34 × 10^7^	−4.384	11,579
	COV	/	3.1%	1.9%	0.1%	10.8%	−0.1%	9.9%
IAM-1	average	1	0.168	0.325	2.1685	1.03 × 10^7^	−4.336	9635
	COV	/	2.5%	1.3%	0.2%	2.4%	−0.1%	3.4%
IAM-2	average	1	0.1615	0.331	2.167	1.09 × 10^7^	−4.334	10,224
	COV	/	0.4%	0.4%	0.1%	5.1%	−0.1%	5.8%

**Table 4:** Interfacial adhesion energy of asphalt binder and mineral fillers.

Mineral Type	E_total_/ (kcal·mol^−1^)	E_asphalt_/ (kcal·mol^−1^)	E_aggragate_/ (kcal·mol^−1^)	A/Å^2^	γ/ (kcal·mol^−1^·Å^−2^)
IT	−189,665	16,310	−205,747	1295	−0.176
LF	−939,906	11,507	−945,939	1288	−4.26

**Table 5:** Reference sequence and comparison sequence.

Sample Types	Fatigue Life	Interfacial Adhesion Energy	Interaction Ability	Complex Modulus
LAM	129,399	1.879	1.37702	4.4564
IAM-1	88,944	0.176	1.85086	5.85565
IAM-2	114,229	0.176	2.35454	7.80645

**Table 6:** Dimensionless data.

Sample Types	Fatigue Life	Interfacial Adhesion Energy	Interaction Ability	Complex Modulus
LAM	1	1	1	1
IAM-1	0.68736	0.09419	1.34410	1.31396
IAM-2	0.88276	0.09419	1.70988	1.75172

**Table 7:** Difference sequence data.

Sample Types	Interfacial Adhesion Energy	Interaction Ability	Complex Modulus
LAM	0	0	0
IAM-1	0.59316	0.65674	0.62662
IAM-2	0.78856	0.82711	0.86897

**Table 8:** Correlation degree of influencing factors of fatigue life.

Sample Types	Interfacial Adhesion Energy	Interaction Ability	Complex Modulus
LAM	1	1	1
IAM-1	0.422	0.398	0.409
IAM-2	0.355	0.344	0.339
Correlation degree	0.592	0.580	0.582

## Data Availability

The original contributions presented in the study are included in the article, further inquiries can be directed to the corresponding authors.
